# Supplementary material for: Comparative Study of the Aftereffect of CO2 Inhalation or Tiletamine–Zolazepam–Xylazine Anesthesia on Laboratory Outbred Rats and Mice
Source: Biomedicines. 2022 Feb 21;10(2):512. doi: 10.3390/biomedicines10020512 (PMC8962436; doi:10.3390/biomedicines10020512)
Supplement: Supplementary file 1 [file biomedicines-10-00512-s001.zip › biomedicines-1552332-supplementary.pdf]

Supplementary Materials:

Comparative Study of the Aftereffect of CO2-Inhalation or Tiletamine-Zolazepam-Xylazine Anesthesia on Laboratory Outbred Rats and Mice

Oksana N. Khokhlova, Natalya A. Borozdina, Elena S. Sadovnikova, Irina A. Pakhomova, Pavel A. Rudenko, Yuliya V. Korolkova, Sergey A. Kozlov, Igor A. Dyachenko

Table S1. Blood glucose level in males and females CD-1 mice in the awake state and after CO2-inhalation or tiletamine-zolazepam-xylazine (TZX) anesthesia

	males				females			
	before CO_2	CO_2	before TZX	TZX	before CO_2	CO_2	before TZX	TZX
	8,1	10,9	6,8	7,7	8,2	9,7	5,4	7,2
	8,7	11,4	8,3	6,7	7,5	8,8	6,3	7,5
	7,6	9,2	8,3	9,8	6,3	11,4	7,8	6,4
	7,2	8,8	7	10,3	6,9	8,1	5,7	8,2
	6,9	11,4	7,9	13,8	7,1	6,1	5,2	8,4
	8	12,7	8,8	7,2	7,3	11,4	6,3	6,1
	7,4	12,4	7,7	8,1	6,3	9,8	6,3	7,1
	6,6	10,6	7,8	10,7	5,9	7,7	6,4	8,1
	7,2	10,3	8,7	11,8	6,4	5,5	6,2	6,8
	8,2	8,2	6,4	7,1	6,8	6,2	7,8	9,3
MEAN	**7,6**	**10,6**	**7,8**	**9,3**	**6,9**	**8,5**	**6,3**	**7,5**
SD	**0,7**	**1,5**	**0,8**	**2,4**	**0,7**	**2,1**	**0,9**	**1,0**

Table S2. Blood glucose level in males and females Sprague-Dawley rats in the awake state and after CO2-inhalation or tiletamine-zolazepam-xylazine (TZX) anesthesia

	males				females			
	before CO_2	CO_2	before TZX	TZX	before CO_2	CO_2	before TZX	TZX
	3,7	4,9	3,7	4,9	4,5	6,6	4,8	6,7
	4,8	7,1	4,5	6,4	5,3	6,2	5,1	5,2
	4,4	4,8	4,6	6,8	5	6,4	4,2	7,3
	4,2	4,3	5,1	6,1	4,4	5,8	3,9	6,4
	4,4	5,3	4,7	6	3,9	5,7	4,6	5,4
	4,4	4,3	4,8	5,8	4,8	5,8	5,3	5,8
	4,2	5,3	4,9	8,3	4,9	7,1	4,2	7,1
	5,6	5,8	4,4	6,7	4,2	5,5	4,2	5,6
	3,8	5,6	4	5,3	4,1	5,6	4,8	6,2
	3,8	4,8	4,2	8,1	4,6	5,9	4,8	5,6
MEAN	**4,3**	**5,2**	**4,5**	**6,4**	**4,6**	**6,1**	**4,6**	**6,1**
SD	**0,6**	**0,8**	**0,4**	**1,1**	**0,4**	**0,5**	**0,5**	**0,7**
